# Supine vs upright exercise in patients with hepatopulmonary syndrome and orthodeoxia: study protocol for a randomized controlled crossover trial

**DOI:** 10.1186/s13063-021-05633-7

**Published:** 2021-10-09

**Authors:** Harsh Parikh, Eric Lui, Marie E. Faughnan, Abdul Al-Hesayen, Stephanie Segovia, Samir Gupta

**Affiliations:** 1 Li Ka Shing Knowledge Institute, Keenan Research Centre for Biomedical Science, St. Michael’s Hospital, Toronto, Canada; 2 Department of Medicine, University of Toronto, Toronto, Canada; 3 Division of Respirology, St. Michael’s Hospital, Toronto, Canada; 4 Division of Cardiology, St Michael’s Hospital, Toronto, Canada

**Keywords:** Hepatopulmonary syndrome, Orthodeoxia, Exercise tolerance, Randomized controlled trials, Liver transplantation

## Abstract

**Background:**

The hepatopulmonary syndrome (HPS) is a pulmonary complication of liver disease found in 10 to 32% of patients with cirrhosis and is characterized by intrapulmonary vascular dilatations and abnormal oxygenation. Liver transplantation is the only effective therapy for this disease. Patients with HPS have significant exercise limitations, impacting their quality of life and associated with poor liver transplant outcomes. Many patients with HPS exhibit orthodeoxia—an improvement in oxygenation in the supine compared to the upright position. We hypothesize that exercise capacity will be superior in the supine compared to the upright position in such patients.

**Methods:**

We propose a randomized controlled crossover trial in patients with moderate HPS (PaO_2_ < 80 mmHg) and orthodeoxia (supine to upright PaO_2_ decrease > 4 mmHg) comparing the effect of supine vs upright position on exercise. Patients with pulmonary hypertension, FEV1/FVC ratio < 0.65, significant coronary artery disease, disorders preventing or contraindicating use of a cycle ergometer, and/or moderate or severe ascites will be excluded. Participants will be randomized to cycle ergometry in either the supine or upright position. After a short washout period (a minimum of 1 day to a maximum of 4 weeks), participants will crossover and perform an exercise in the alternate position. Exercise will be performed at a constant work rate of 70–85% of the predicted peak work rate until the “stopping time” is reached, defined by exhaustion, profound desaturation, or safety concerns (drop in systolic blood pressure or life-threatening arrhythmia). The primary outcome will be the difference in the stopping time between exercise positions, compared with a repeated measures analysis of variance method with a mixed effects model approach. The model will be adjusted for period effects. *P* < 0.05 will be considered statistically significant.

**Discussion:**

HPS patients have hypoxemia leading to significant exercise limitations. If our study is positive, a supine exercise regimen could become a routine prescription for patients with HPS and orthodeoxia, enabling them to exercise more effectively. Future studies could explore the corresponding effects of a supine exercise training regimen on physiologic variables such as long-term exercise capacity, quality of life, dyspnea, and liver transplantation outcomes.

**Trial registration:**

ClinicalTrials.gov Protocol Registration and Results System (PRS) NCT04004104. Registered on 1 July 2019

**Supplementary Information:**

The online version contains supplementary material available at 10.1186/s13063-021-05633-7.

## Background

The hepatopulmonary syndrome (HPS) is a pulmonary complication of liver disease found in 10 to 32% of patients with cirrhosis [1]. It is defined by the combination of [1] liver dysfunction or portal hypertension, [2] intrapulmonary vascular dilatations, and [3] abnormal oxygenation [2]. Liver transplantation is the only known effective therapy for this disease [3].

### Exercise in the hepatopulmonary syndrome

Participants with liver disease have reduced exercise capacity compared to normal controls [measured by peak oxygen consumption (VO_2peak_)] [4, 5] due to a combination of deconditioning, malnutrition-associated muscle weakness, and anemia [6]. Exercise tolerance is further impaired in patients with HPS [7–9], who have more dyspnea and a reduced New York Heart Association functional class, compared to patients with cirrhosis who do not have HPS [10]. Formal exercise testing data are available in four small reports and one large cross-sectional study and demonstrate reduced exercise capacity and profound exercise desaturation in HPS (Table 1). The authors have hypothesized that this exercise desaturation is the result of increased shunt physiology, worsening diffusion due to increased pulmonary blood flow with reduced capillary transit time (a physiologic phenomenon called the “diffusion-perfusion defect”), and a reduced mixed venous oxygen content, the impact of which on arterial oxygen saturation is magnified by the former two effects [9]. These studies support the concept that an abnormal pulmonary circulation contributes to exercise limitation in HPS and that patients with HPS experience severely reduced aerobic capacity, beyond that found in those with cirrhosis without HPS [7–9, 11, 12].

**Table 1:** Previous literature reporting exercise testing in patients with HPS

Study		Exercise findings
Thorens et al., 1992 [11]	HPS case report (*n* = 1); constant work rate test*	• Worsening physiologic shunt (from 12% at rest to 26% with exercise)
Epstein et al., 1998 [7]	HPS (*n* = 5) vs cirrhosis^†^ (*n* = 19); incremental cycle ergometry	• Reduced VO_2peak_ (55% predicted in HPS vs 72% predicted in cirrhosis) • Progressive exercise hypoxemia • Earlier onset of the anaerobic threshold • Elevated dead space ventilation
Whyte et al., 1998 [9]	HPS (*n* = 8); incremental cycle ergometry	• Progressive exercise desaturation • Diminished achieved workload (mean 48% predicted) • Reduced mixed venous oxygen content
Nusair et al., 2005 [8]	HPS case report (*n* = 1); incremental cycle ergometry	• Reduced VO_2peak_ (41% predicted) • Progressive exercise hypoxemia • Marked dyspnea • Worsening physiologic shunt
Faustini-Pereira et al., 2015 [12]	HPS (*n* = 92) vs cirrhosis^‡^ (*n* = 86); modified Bruce protocol*	• Reduced VO_2peak_ (80.2% predicted in HPS vs 86.7% predicted in cirrhosis) • Reduced 6-min walk distance (341 m in HPS vs 416 m in cirrhosis)

*HPS* hepatopulmonary syndrome, *VO*_*2peak*_ maximum rate of oxygen consumption measured during incremental exercise, *m* meters

*Exercise modality not specified

^†^“Cirrhosis” defined as PaO_2_ ≥ 90 mmHg and alveolar-arterial oxygen gradient < 20 mmHg (negative contrast echo not required)

^‡^“Cirrhosis” defined as alveolar-arterial oxygen gradient < 20 mmHg (negative contrast echo not required)

### Orthodeoxia in the hepatopulmonary syndrome

Intrapulmonary vascular dilatations (IPVDs) are believed to cause the hypoxemia of HPS through a “diffusion-perfusion defect” [11]. This is a combination of an increased distance between the alveolar membrane and the red blood cells in the center of dilated capillaries, causing an effective diffusion abnormality, along with a reduced resistance to flow causing increased perfusion through the dilated capillaries—which reduces available time for equilibration between the alveolar gas and the blood [13]. These IPVDs are often most prominent at lung bases [14]. Accordingly, due to the gravitational redistribution of blood flow to the lung bases in the upright position, there is an increase in blood volume passing through IPVDs, resulting in a worsening diffusion-perfusion defect when moving from the supine to the upright position [15]. A corresponding drop in partial pressure of arterial oxygen (PaO_2_) of greater than 5% or 4 mmHg in the upright compared to the supine position is called orthodeoxia [1]. This is often associated with a perception of increased dyspnea when upright called platypnea [14].

### Study rationale and purpose

Limited current physiologic data suggest an important role for hypoxemia in the exercise limitation caused by HPS, suggesting that HPS patients with orthodeoxia may have a greater exercise capacity when exercising in the supine position compared to the conventional upright position. Previous studies have compared upright to supine exercise in various populations. In healthy individuals, although cardiac output increases in the supine exercise due to an increased preload and stroke volume [16–18], there is also reduced blood flow to the leg muscles [19], resulting in reduced muscle oxygen uptake, more profound muscle deoxygenation [20], and a lower anaerobic threshold [19] compared to the upright exercise. In patients with comorbidities, supine exercise has generally been found to worsen physiologic parameters compared to upright exercise, including a drop in alveolar ventilation (with an increase in partial pressure of end tidal CO_2_) [21] in patients with chronic obstructive pulmonary disease (COPD), a failure to increase left ventricular ejection fraction in patients with hypertension [22], and ST segment depression possibly indicating a lower ischemic threshold in patients with coronary artery disease [23]. However, positional effects on exercise in patients with HPS, and the unique impact of orthodeoxia have not been reported.

The primary aim of our study is to evaluate the effect of the supine position on exercise in HPS participants with orthodeoxia, compared to exercise in the upright position.

## Methods and design

### Objective and hypothesis

Our primary objective is to study the effect of supine position, compared to the upright position, on exercise parameters in participants with HPS and orthodeoxia. We hypothesize that these participants will have improved exercise time when supine, compared to upright.

### Trial design

**Fig. 1:**
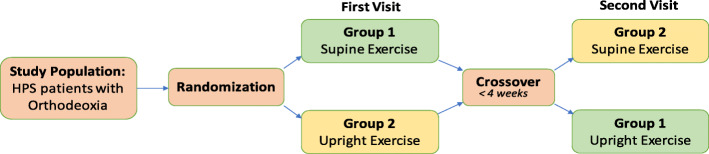
Study design

This will be a randomized controlled crossover trial, conducted in the Canadian HPS Program [an HPS clinical and research program founded in 2005 (www.hpscare.com), consisting of sites in Toronto, Ontario (Unity Health Toronto and University Health Network), and Montreal, Quebec (Centre hospitalier de l’Université de Montréal)]. Eligible participants will be identified by clinicians in the Canadian HPS Program through the existing Canadian HPS Program Database (a database of all HPS patients seen in the Canadian HPS Program who have consented for data collection and to be approached for future research). Once patients have provided assent to the clinician, they will be contacted by independent research personnel, either by phone or in-person during clinic visits, to seek informed consent (see Additional file 1 for the consent form). Consenting patients will be randomized to start with either a supine or upright exercise test on a bicycle ergometer. Subsequently, participants will complete the alternate test on a separate day, within 4 weeks of the first test (Fig. 1). The allocation sequence will be generated by a research coordinator in advance, through a computerized random number generator. This research coordinator will assign the testing order for each newly recruited participant, and only this person will have access to the allocation sequence, which will remain concealed from study investigators at all times.

### Participants

Participants will be sequentially recruited from the Canadian HPS Program Database between late 2019 and approximately mid-2023 (including a pause due to SARS-COV-2 for much of 2020). The eligibility criteria are outlined in Table 2. The drop-out criteria are provided under the “Exercise protocol” section.

**Table 2:** Eligibility criteria

**Inclusion criteria** 1. Moderate HPS: (a) Liver disease (evidence of synthetic liver dysfunction and/or portal hypertension on biochemistry and/or imaging) (b) Moderate hypoxemia ▪ PaO_2_ < 80 mmHg ▪ AaDO_2_ ≥ 15 mmHg or ≥ 20 mmHg if age > 64 [1] (c) Intrapulmonary vascular dilatations (microbubbles seen in the left heart ≥ 3 cycles after the right heart on saline contrast echocardiography) 2. Presence of orthodeoxia (PaO_2_ decrease by > 4 mmHg when the patient moves from supine to upright position) **Exclusion criteria** 1. Pulmonary hypertension (a) Echocardiographic estimated right ventricular systolic pressure ≥ 50 mmHg (b) Right heart catheterization mean pulmonary artery pressure > 25 mmHg with pulmonary capillary wedge pressure ≤ 15 mmHg 2. Significant obstructive ventilatory impairment (FEV1/FVC ratio < 0.65) [14] 3. Known significant coronary artery disease 4. Significant neurologic, orthopedic, or rheumatological disorders preventing the use of a cycle ergometer 5. Other absolute contraindications to submaximal exercise testing [24] (a) Uncontrolled cardiac arrhythmia with hemodynamic compromise (b) Symptomatic severe aortic stenosis (c) Decompensated heart failure (d) Acute cardiopulmonary illness (e.g., venous thromboembolism, myocarditis, pericarditis, endocarditis, acute aortic dissection) 6. Moderate or severe ascites	

*PaO*_*2*_ partial pressure of arterial oxygen, *FEV1/FVC* forced expiratory volume in 1 s over forced vital capacity, *AaDO*_*2*_ alveolar-arterial oxygen gradient [PAO_2_ − PaO_2_, where PAO_2_ = [FiO_2_ (*P*_atm_ − *P*_H2O_) − PaCO_2_/0.8]], *AO*_*2*_ partial pressure of alveolar oxygen, *FIO*_*2*_ inspiratory oxygen fraction, *P*_*atm*_ atmosphere pressure, *P*_*H2O*_ water vapor partial pressure, *PaCO*_*2*_ arterial carbon dioxide pressure

### Exercise protocol

Each participant will perform a constant work rate exercise test (CWRET). The constant work rate will be individualized for each participant and set at 70–85% of their estimated peak work rate [25], targeting an estimated test duration of 180–480 s (to increase the chances that exercise limitation is due to the physiologic effects of exercise rather than physical discomfort or boredom [26]). Peak work rate will be estimated from a previous room air 6-min walk test (6MWT) performed within the last 6 months (peak work rate = 0.168 × 6MWD (m) − 4.085) [27].

The CWRET will include a warmup period consisting of 1 min of rest during which participants will be in set position with their feet planted on the pedals, without pedaling. This will be followed by an immediate ramp-up to the predetermined target constant work rate [25, 26]. Patients will be instructed to pedal at a rate between 50 and 60 revolutions per minute (rpm) and will be provided with constant feedback on pedaling frequency through a biofeedback display. We will also provide standardized verbal encouragement throughout [28], relating when they are above or below the target pedaling frequency, congratulating them when they are within the target frequency, and encouraging them to continue pedaling for as long as they feel able to. For each participant, exercise in each position will be standardized with respect to the proper seat adjustment relative to leg length and pedaling cadence. Upright cycle ergometry will be performed on the Corival Ergometer Bicycle (LODE B.V. Medical Technology Groningen, the Netherlands) at an angle of 90°. The supine cycle will be performed on the Stress EchoBed® (Medical Positioning Inc., USA) at an angle of 0°. For both tests, all measurements will take place through a pitot tube spirometer. Volume and flow calibrations (pitot tube spirometer) and gas calibration (Ergo Card Analyzer, Medisoft, USA) will be performed within 1 h of the exercise test, and Bio calibration will be performed on the pitot tube spirometer every 3 months.

Inspiratory capacity will be measured before and after exercise. Before, after, and throughout exercise, we will measure the following: oxygen uptake (VO_2_) and carbon dioxide production (VCO_2_) (measured breath-by-breath, averaged over 30-s epochs), oxygen saturation and heart rate (continuously, by pulse oximetry and 12-lead electrocardiography, respectively), blood pressure (every 2 min, by manual sphygmomanometry), and subjective dyspnea and leg fatigue (every 1 min, by modified Borg scale) [29]. Participants will continue exercising until they reach one of the following stopping criteria: 1) the point at which, after standardized encouragement, the participant is unable to continue because of symptoms (i.e., participant does not wish to continue or is unable to maintain a minimum peddling frequency of 40 rpm for ≥ 10 s) [defined as the “tolerable limit” (tLIM)]; 2) desaturation below a set point for ≥ 30 s; 3) a drop in systolic blood pressure by ≥ 10 mmHg from baseline; or 4) the appearance of life-threatening arrhythmias (such as significant ventricular arrhythmias or high-grade heart block). The low saturation set point will be chosen individually for each participant, as the lower of 80% [26], or the nadir desaturation seen on baseline room air 6MWT. Stopping time will be defined as the duration of pedaling during the constant workload exercise test before a stopping criterion was met. During the test, the stopping time and criterion will be determined by agreement between the PI and the experienced technician conducting the test. Upon agreement, the time and criterion will be designated within the CPET software and independently recorded on a data collection sheet by a research assistant (the assistant will also use a stopwatch during the test to independently record timing, ensuring that stopping time is measured in duplicate to account for any system failures or errors). The dropout criteria for this study include unwillingness/inability to return for the second test or presence of one of the arrhythmic or hemodynamic-related stopping criteria on the first of the protocolized exercise tests (a safety-related dropout).

Participants will be reminded to bring running shoes and comfortable exercise clothes, to ensure that they have eaten before the test, to take all usual medications, and to avoid major exercise for 24 h before the test. A physician with expertise in cardiopulmonary exercise testing will be in attendance for monitoring throughout test procedures. Given the small sample size and the high relative safety of the exercise intervention, we will not be performing official interim analyses or creating a data and safety monitoring board. However, if any safety event occurs during study conduct, the investigators will immediately re-evaluate the safety of the protocol and overall study. Upon completion, patients will not only be notified of the overall study results and publication, but also of their individual performance and whether supine exercise was found to be beneficial in their case.

### Outcome measures

Exercise tests will be analyzed by a pulmonologist with experience in cardiopulmonary exercise test interpretation. This assessor will be masked to exercise position.

### Primary outcome measure

The primary outcome will be the difference in stopping time between the upright and supine exercise positions. We will exclude participants who stopped the exercise test due to either life-threatening arrhythmia or a drop in systolic blood pressure from the primary outcome analysis.

### Secondary outcome measures

Secondary outcomes will include differences in the following variables at isotime: oxygen uptake (VO_2_), minute ventilation (VE), work rate, heart rate (HR), arterial oxygen saturation (SpO_2_), dyspnea, leg fatigue, change in inspiratory capacity, and carbon dioxide production (VCO_2_). We will also compare the reason for stopping exercise (leg fatigue, dyspnea, other) and maximum minute ventilation (*VE*_max_). In patients who reach anaerobic threshold (AT) in both positions, we will compare time to reach AT, and VO_2_, VE/VCO_2_, and cardiac output at AT in each position. Relationships between key variables will be compared graphically, including VCO_2_ over VO_2_, HR over VO_2_,VE over VCO_2_, end-tidal CO_2_ (PetCO_2_) over time, saturation over time, VE over time, and VO_2_/HR (“oxygen pulse”) over time and HR over time. We will also conduct exploratory subgroup analyses, investigating the effects of baseline values such as PaO_2_ and degree of orthodeoxia on exercise variables.

### Recruitment and power

Given that HPS is a rare disease, and a majority of patients progress to either liver transplant or death relatively soon after diagnosis [10], recruitment to prospective trials in HPS has previously proven very challenging [30, 31]. Our recruitment will be further limited by the fact that only a subset of patients with HPS has orthodeoxia. Accordingly, we first estimated a recruitment target based on feasibility then set out to determine whether the demonstrable effect size with this sample would be both plausible and clinically meaningful. To estimate feasible recruitment, we searched the literature for studies describing the prevalence of orthodeoxia in cohorts of ≥ 10 HPS patients. However, we found only 4 small reports (14–20 patients each) reporting a highly variable prevalence of orthodeoxia, ranging from 14 to 88% of HPS participants (Table 3).

**Table 3:** Characteristics of orthodeoxia in patients with hepatopulmonary syndrome

Author	Mean upright^†^ PaO_**2**_ (mmHg)	Prevalence of orthodeoxia^‡^, ***N*** (%)	Mean room air PaO_**2**_ in patients with orthodeoxia (mmHg) (SD)	Orthodeoxia (supine PaO_**2**_ − upright PaO_**2**_; mmHg)
Krowka et al., 1993 [32]	44.0	14/16 (87.5)	Upright: 44.0 ± 9 Supine: 62.0 ± 14	18.0
Martinez et al., 1999 [33]	65.2	2/5 (40.0)	Upright: 51.0 ± 3 Supine: 61.5 ± 2	10.5
Martinez et al., 2001 [34]	75.0	2/14 (14.3)	Upright: 63.5 ± 21 Supine: 71.5 ± 21	8.0
Gomez et al., 2004 [35]	69.0	5/20 (25.0)	Upright: 59.0 ± 6 Supine: 67.0 ± 5	8.0
Current study*	51.8	37/56 (66.1)	Upright: 48.1 ± 14 Supine: 61.9 ± 12	13.8

*Analysis performed in patients at the Toronto site of the Canadian HPS Database with a PaO_2_ < 80 mmHg, absence of significant concurrent lung disease contributing to hypoxemia, and absence of concurrent portopulmonary hypertension

^†^All studies defined “upright” as the sitting position, except for Krowka 1993 [32] and the current study, which defined it as the standing position

^‡^All studies defined orthodeoxia as a drop in partial pressure of arterial oxygen (PaO_2_) of greater than 5% or 4 mmHg in the upright compared to the supine position, except Martinez 1999 [33], which defined it as a drop in PaO_2_ greater than 10 mmHg in the upright compared to the supine position

We hypothesized that these variations in prevalence may have been due to the differences in covariates which predict orthodeoxia between study populations; however, the only study to attempt to explore predictors of orthodeoxia was that by Gomez and colleagues, in which the only significant predictors of orthodeoxia in a cohort of 20 patients with HPS were a lower baseline cardiac index and higher mean distribution of upright alveolar ventilation. Factors including baseline PaO_2_, etiology of liver disease, age, VO_2_, minute ventilation, and diffusion lung capacity of carbon monoxide (DLCO) were not significant predictors [35].

Given these limited sample sizes upon which to base our estimates, we performed an analysis of the prevalence and predictors of orthodeoxia in patients in the Canadian HPS Program Database. A priori, we identified the following candidate baseline predictors: age, sex, PaO_2_, MELD score, DLCO, macroaggregated albumin (MAA) shunt fraction, 6MWD, and presence of clubbing. We found that 37 out of 56 patients (66%) had orthodeoxia. In univariate analyses, lower baseline upright PaO_2_ and DLCO were significant predictors of orthodeoxia in HPS (Table 4).

**Table 4:** Differences in baseline clinical characteristics between hepatopulmonary syndrome patients with and without orthodeoxia (current cohort)

Characteristic		No orthodeoxia	Orthodeoxia	***P*** value^†^
Age (years)	*N*	19	37	
	Mean ± *SD*	63.8 ± 11.9	60.4 ± 10.7	0.302
Sex—male	*N* (%)	8 (44.4)	22 (59.5)	0.294
MELD score	*N*	17	36	
	Mean ± *SD*	14.0 ± 3.8	12.4 ± 3.4	0.144
DLCO (% predicted)	*N*	18	35	
	Mean ± *SD*	64.0 ± 14.7	50.2 ± 14.7	0.005
MAA shunt fraction (%) [36]	*N*	10	30	
	Mean ± *SD*	17.2 ± 23.2	22.0 ± 13.8	0.551
Shunt fraction on 100% FiO_2_ (%) [37]	*N*	19	34	
	Mean ± *SD*	13.4 ± 6.0	14.6 ± 8.3	0.571
6MWD (m)	*N*	7	10	
	Mean ± *SD*	418.6 ± 109.2	441.8 ± 147.8	0.715
Clubbing	*N* (%)	12 (63.2)	22 (61.1)	0.882
PaO_2_ (upright) (mmHg)	*N*	19	37	
	Mean ± *SD*	59.0 ± 13.0	48.1 ± 14.3	0.006

*MELD* denotes model for end-stage liver disease score, *DLCO* diffusion lung capacity for carbon monoxide, *MAA* macroaggregated albumin, *FiO*_*2*_ fraction of inspired oxygen, *6MWD* 6-min walk distance, *PaO*_*2*_ partial pressure of arterial oxygen, *N* total number of participants, *SD* standard deviation

^†^Univariate analyses are presented for each variable, continuous variables assessed with a 2-sample *t*-test, and categorical variables with a chi-squared test. Variables were assessed on the same day as orthodeoxia or within an interval of ≤ 1 year

To our knowledge, this is by far the largest analysis of both the prevalence and risk factors for orthodeoxia in HPS. Given that patients with orthodeoxia had more severe hypoxemia, it was not surprising that our more “severe” HPS cohort (compared to other reports) had a high observed orthodeoxia prevalence of 66.1% (Table 3). We complimented this with an analysis of our current active HPS database, revealing that six patients currently meet the inclusion criteria. Additionally, a review of referrals in the last 3 years reveals that an average of 4 eligible patients is referred to our program each year. Based on a recruitment target of 50%, this analysis of the Canadian HPS Program Database suggests that we will be able to recruit 10 eligible participants to this study in the 4-year recruitment window allowed by study funding. We believe that this target is achievable, particularly with our pessimistic 50% recruitment target, since patients with HPS and orthodeoxia have no effective treatment options other than liver transplant, the study involves nearly no risk, and the study offers an opportunity to identify a clinically beneficial exercise strategy for the individual.

A crossover design has previously been successfully employed in patients with HPS [30]. The advantage of the crossover design in rare diseases such as HPS is that each participant will undergo both interventions, and within-person comparisons will limit confounding and reduce inter-subject variability, thereby reducing the sample size required to demonstrate an effect [38]. The crossover design is well-suited to an exercise intervention because there is no anticipated therapeutic carryover effect, obviating the need for a washout (we allowed for a 1-day minimum “washout” period for recovery from the prior exercise test). We established a 4-week maximum period between tests to minimize any possible period effect (i.e., to prevent significant disease progression by the time of the second test). The possibility of a period effect due to familiarity with the cycling exercise will be evaluated statistically.

Calculating the demonstrable effect size in this sample (10 participants) requires an estimate of the standard deviation of the expected change in stopping time between supine and upright positions. However, due to the novelty of this study design, there is no existing literature investigating supine exercise in patients with HPS. We also did not find any studies comparing supine and upright exercise in patients with cirrhosis without HPS. However, we did identify a study that employed a crossover design to evaluate the effect of hyperoxia (which has a similar physiologic impact to supine position in our cohort) on CWRET stopping time [39], in patients with interstitial lung disease—a condition in which the primary abnormality is a reduced diffusion capacity, which may have a comparable physiologic impact on exercise as the diffusion-perfusion defect of HPS. This study showed that exercise time increases significantly with hyperoxia compared to room air (21.9 ± 12.9 vs 11.6 ± 10.0 min, *P* < 0.001) [39], with a pooled standard deviation for change in exercise duration of 11.5 min. Applying this standard deviation, our crossover trial with a target sample size of 10 participants will be able to detect a difference of ≥ 2.85 min between the two interventions with power of 80% and a two-sided alpha of 0.05.

An increase in exercise time of 2.85 min is clinically meaningful in other hypoxemic diseases. In COPD, the minimal clinically important difference (MCID) for tLIM on CWRET is an increase of 33% or 105 s from baseline [26]. Bronchodilator trials suggest that clinical outcome improvements correspond to tLIM improvements of > 60 s [26]. Accordingly, an improvement of 2.85 min (171 s) would likely be clinically significant in our participants, who have more severe baseline exercise limitations than typical patients with COPD. With an estimated upright test duration of 3–8 min in our design [26], a change of 2.85 min would represent an improvement of between 36 and 95%, which again suggests a clinically meaningful improvement.

### Auditing and data management

Each step of the study process, including test conduct, data recording, and data analysis, will be conducted through the guidance of standard operating procedures (SOPs). A research coordinator and the PI will be present at each exercise study to audit and ensure compliance of the test conduct and data recording with SOPs. A linking log will be used to track the recruitment of participants into the study. Participant personal health information (PHI) such as full name, date of birth, MRN, email, and phone number will be collected and stored within the master linking log. All participants will be assigned a study ID (matching their Canadian HPS Program Database ID), which will be used alongside the rest of the results. The linking log will be separately and securely stored on an institutional drive for 5 years post-study closure, after which it will be destroyed. Only designated research personnel will have access to the data. The de-identified dataset will be stored for a period of 7 years after study completion.

### Ancillary and post-trial care

In the unlikely event of research-related side effects or injury, medical care will be provided by the research physician at no charge, and/or the participant will be referred for required medical care. Although no funds have been set aside to compensate the participant in the event of injury or illness related to the study procedures, the participant does not give up any legal rights for compensation by consenting to and participating in this study. The investigator, the hospital, the sponsor, and participants’ agents are not relieved from their legal and professional responsibilities.

### Statistical analysis

Continuous variables will be reported as mean (median) ± standard deviation, and categorical variables will be reported as proportions or percentages. We will employ a repeated measures analysis of variance method with a mixed effects model approach to compare the primary and secondary outcomes between interventions. The model will be adjusted for the period in which the treatment was received to assess for the period effect. An interaction between treatment and period will be included to account for the carryover effect. Relationships between variables and between baseline characteristics and exercise test results will be explored with parametric or non-parametric tests of correlation, as appropriate. The normality of continuous variables will be evaluated using QQ plots and histograms. Model residuals will be assessed graphically to ensure that the model satisfies the normality and constant variance assumptions. The outcome will be log-transformed to stabilize the variable, and possible correlation structures will be employed if the assumptions are found to be violated. We will also test for period and carryover effects. The significance level (*α*) will be set at < 0.05. All analyses will be conducted in the R software, version 4.0.3 [40].

## Discussion

Our study aims to investigate the effect of position change on exercise capacity in HPS patients with orthodeoxia. This will be the first study to describe exercise capacity in the supine position in HPS, the first to compare with upright exercise, and the first to describe the use of a CWRET protocol in a cohort with this disease. Given the novelty of our research question and approach, the development of this study posed a number of unique challenges and opportunities which merit discussion.

### Study population and design

We chose to include patients with at least moderate HPS (PaO_2_ < 80 mmHg), in order to ensure that the degree of observed orthodeoxia is both clinically and physiologically significant. Tissue oxygen delivery (DO_2_), which is the physiologic substrate for the hypothesized position-related changes in exercise capacity that we seek to demonstrate, is dependent on oxygen saturation, which is in turn correlated with PaO_2_ through the sigmoidal oxyhemoglobin dissociation curve. Given the flat shape of this curve at higher PaO_2_ levels, changes in PaO_2_ of just over 4 mmHg (the definition of orthodeoxia) would not result in any significant changes in oxygen saturation in patients with a baseline PaO2 ≥ 80 mmHg.

Our use of a CWRET protocol is novel in this population, as prior studies of exercise testing in HPS (Table 1) have almost exclusively employed incremental exercise protocols [7–9, 12]. Given the severe baseline disease in our expected patient population (Table 3), with a predicted mean PaO_2_ drop of 20 mmHg at peak exercise [7], we believe that an incremental exercise protocol would result in profound desaturation requiring cessation for safety reasons, with a high resulting likelihood of a submaximal test. Accordingly, the main variable of interest in an incremental exercise protocol, VO_2peak_, would not likely be achieved in most participants. To address this, we chose a high-intensity CWRET, which has been widely used to assess the changes in exercise tolerance following interventions in other chronic hypoxemic lung diseases [26].

The constant work rate in a CWRET is typically set at 70–85% of the peak work rate measured on incremental exercise testing (IET) [26]. Given that an IET was not feasible in our population, we instead adopted a validated prediction equation for estimating peak work rate based on 6-min walk distance (6MWD) in patients with COPD [27, 41]. Given that peak work rate is affected by ventilatory impairment and dynamic hyperinflation in COPD, neither of which appears to play a role in HPS, this represents a vulnerability in our testing protocol.

### Safety

The primary outcome in our study is stopping time, as determined by either reaching tLIM or experiencing desaturation to our pre-set safety stopping criterion, which is the lower of 80% or the nadir desaturation seen on room air 6MWT. There have been no reports of adverse events while performing CWRET [26]. While there is no definitive threshold at which arterial desaturation becomes hazardous [26], cardiopulmonary exercise testing (CPET) guidelines from the American Thoracic Society and American College of Chest Physicians suggest a saturation of ≤ 80% (with accompanying signs and symptoms of severe hypoxemia) as one of the indications for exercise termination [42, 43]. However, patients with HPS are well adapted to hypoxemia [1]. These patients often present after a prolonged period of undiagnosed hypoxemia and are encouraged to exercise to preserve muscle mass despite significant exercise desaturation (which occurs even with oxygen supplementation) [44, 45]. Furthermore, many patients who require oxygen do not use it at all times [46]. Accordingly, and because many patients in our severe population would desaturate to 80% at rest or with minimal exertion while upright, we added an individualized stopping criterion set at the nadir desaturation experienced on room air 6MWT. This novel approach will enable patients with severe disease to perform a sufficient amount of exercise for positional differences to be detectable and given that it likely reflects a level of desaturation that patients typically experience in their daily lives, and will maintain a reasonable margin of safety.

### Clinical relevance of the potential results

Exercise has been shown to have numerous health benefits, ranging from reducing the risk of heart disease, stroke, osteoporosis, diabetes, and cancer to improving mental health [47]. Unfortunately, patients with HPS are unable to realize the short- or long-term (training) benefits of exercise due to severe exercise limitations caused by hypoxemia. If our hypothesis proves correct, a supine exercise protocol would empower these patients to exercise for longer periods of time. Furthermore, supine exercise could enable a more effective long-term exercise training program. Exercise training results in improvements in exercise capacity, including maximum oxygen uptake (VO_2max_), VO_2peak_, and muscle mass in patients with cirrhosis [4, 48, 49]. This could be particularly impactful for patients with HPS awaiting liver transplant, given that this is the only treatment for HPS, and that pre-transplant exercise capacity predicts post-transplant survival [50, 51]. Every 100-m increase in baseline 6MWD is associated with a 52% reduction in 1-year post-transplant mortality [52], and preoperative exercise capacity independently predicts respiratory complications post-liver transplant [53]. Furthermore, the median liver transplant wait time in severe HPS is 200 days [54], and these patients experience progressive hypoxemia [3, 44], resulting in worsening exercise limitation while awaiting transplant. It is also of note that the HPS patients being targeted by this intervention—those with orthodeoxia—tend to have a lower baseline PaO_2_ and are thus more likely to require a liver transplant for HPS.

If positive, this study will establish the efficacy of supine exercise in this population, enabling this to become a routine part of HPS management, including in non-transplant and pre-transplant settings. This evidence would be required to justify the cost and complexity of a supine exercise prescription. Our results would warrant future studies investigating the long-term physiologic and clinical benefits of a supine exercise training program in HPS, including effects on patient-relevant outcomes such as quality of life, dyspnea, and liver transplant outcomes. Our data suggest that two-thirds of patients with at least moderate HPS have orthodeoxia, representing a significant population of patients (particularly, those with the most severe disease) that could stand to benefit. We also believe that findings from our unique exercise protocol will advance understanding of the physiology of this disease and lay the foundations for larger future studies.

### Limitations

It is important to note that our findings will only be applicable to patients with HPS who have orthodeoxia. As noted, 66% of patients with at least moderate HPS had orthodeoxia in our cohort, but smaller studies have reported an orthodeoxia prevalence as low as 14% [34] in all-comers with HPS, and this requires further study. We also note that while orthodeoxia is measured in the supine vs the standing position, the upright bicycle exercise protocol more closely simulates sitting than standing, and prior reports have suggested that orthodeoxia is less pronounced in the sitting compared to the standing position [55]. A smaller positional change in PaO_2_ could reduce the predicted effect of position on exercise capacity.

## Trial status

Protocol version #3, version date 27 August 2019. Enrollment into the trial has started and is expected to be finalized by approximately July 1, 2023.

## Supplementary Information

**Additional file 1: Table S1.** HPS Exercise Protocol Schedule of Forms and Procedures.

## Data Availability

The datasets generated and/or analyzed during the current study will be made available by the corresponding author to fellow investigators, upon reasonable request and after approval by the institutional research ethics board. There are no contractual relationships limiting anonymous data sharing.

## Abbreviations

HPS: Hepatopulmonary syndrome
VO_2peak_: Peak oxygen consumption
IPVDs: Intrapulmonary vascular dilatations
PaO_2_: Partial pressure of arterial oxygen
COPD: Chronic obstructive pulmonary disease
FEV1/FVC: Forced expiratory volume in 1 s over forced vital capacity
AaDO2: Alveolar-arterial oxygen gradient
AO_2_: Partial pressure of alveolar oxygen
*P*_atm_: Atmosphere pressure
*P*_H2O_: Water vapor partial pressure
PaCO_2_: Arterial carbon dioxide pressure
CWRET: Constant work rate exercise test
6MWT: Six-minute walk test
RPM: Revolutions per minute
tLIM: Tolerable limit
VO_2_: Oxygen uptake
VE: Minute ventilation
HR: Heart rate
SpO_2_: Arterial oxygen saturation
VCO_2_: Carbon dioxide production
*VE*_max_: Maximum minute ventilation
AT: Anaerobic threshold
DLCO: Diffusion lung capacity of carbon monoxide
MELD: Model for end-stage liver disease score
MAA: Macroaggregated albumin
FiO_2_: Fraction of inspired oxygen
6MWD: Six-minute walk distance
SD: Standard deviation
MCID: Minimal clinically important difference
DO_2_: Tissue oxygen delivery
IET: Incremental exercise testing
CPET: Cardiopulmonary exercise testing
VO_2max_: Maximum oxygen uptake
