# Supplementary material for: Supine vs upright exercise in patients with hepatopulmonary syndrome and orthodeoxia: study protocol for a randomized controlled crossover trial
Source: Trials. 2021 Oct 9;22:683. doi: 10.1186/s13063-021-05633-7 (PMC8500814; doi:10.1186/s13063-021-05633-7)
Supplement: Supplementary file 1 — Additional file 1: Table S1. HPS Exercise Protocol Schedule of Forms and Procedures. [file 13063_2021_5633_MOESM1_ESM.doc]

Appendix Table 1. HPS Exercise Protocol Schedule of Forms and Procedures

|  | **STUDY PERIOD** | | | | |
| --- | --- | --- | --- | --- | --- |
|  | **Enrolment** | **Allocation** | **Post-allocation** | | |
| **TIMEPOINT** | ***-t_1_*** | **0** | ***t_1_*** | ***t_2_*** | ***t_3_*** |
|  | ***Enrolment/Screening/Consent*** | **Baseline/**  **Randomization** | ***First visit*** | ***Cross-over***  ***(< 4 weeks)*** | ***Second visit*** |
| **ENROLLMENT:** |  |  |  |  |  |
| **Eligibility screen** | X |  |  |  |  |
| **Informed consent** | X |  |  |  |  |
| ***Randomization*** |  | X |  |  |  |
| **INTERVENTIONS:** |  |  |  |  |  |
| **Group 1** |  |  |  |  |  |
| ***Supine Exercise*** |  |  | X |  |  |
| ***Upright Exercise*** |  |  |  |  | X |
| **Group 2** |  |  |  |  |  |
| ***Supine Exercise*** |  |  |  |  | X |
| ***Upright Exercise*** |  |  | X |  |  |
| **ASSESSMENTS:** |  |  |  |  |  |
| ***Inspiratory Capacity*** |  |  | X |  | X |
| ***Cardiopulmonary Exercise Test parameters:***   - ***oxygen uptake (VO2)*** - ***carbon dioxide production (VCO2)*** - ***oxygen saturation*** - ***heart rate*** - ***12-lead electrocardiography*** - ***blood pressure*** - ***stopping reason and time*** |  |  | X |  | X |
| ***Borg scale*** |  |  | X |  | X |
